# Evaluating the Impact of Digital Support on Parental Stress in Swedish Child Health Care: Results From an Intervention Study

**DOI:** 10.1155/ijpe/8780069

**Published:** 2025-06-24

**Authors:** Lotha Valan, Ulf Isaksson, Asa Hörnsten, Asa Carlsund

**Affiliations:** ^1^Department of Nursing, Mid Sweden University, Sundsvall, Sweden; ^2^Department of Nursing, Umea University, Umea, Sweden

## Abstract

**Introduction:** The Swedish child health care (CHC) program provides voluntarily, at no cost, services for children from birth to 5 years old. Participation rates are 99% of Swedish parents enrolling their children in some form of CHC program. Parental groups, comprising parents with similar experiences, can help reduce parental stress and foster the development of effective coping strategies. The study is aimed at evaluating a digital support intervention involving parents, child health nurses, and researchers.

**Methods:** This cluster-randomized, prospective pilot intervention study, conducted in northern Sweden, had three follow-up points: baseline, 4 months, and 8 months. Data were collected from autumn 2022 to late spring 2023 and evaluated effects on parental stress and satisfaction, eHealth literacy, and satisfaction with CHC, accessibility, and support. The 18-item Parental Stress Scale was used to assess parental stress and satisfaction. eHealth literacy was measured using the 10-item eHEALS scale, and parental satisfaction and opinions on accessibility to CHC were measured using a three-item Visual Analogue Scale. The intervention group was offered to participate in various digital activities, while the control group received the usual CHC.

**Results:** Parental satisfaction and stress levels within and between the intervention and control groups showed no significant changes from baseline to 8 months. Regarding eHealth literacy, differences were observed between the groups; however, both groups demonstrated improvement at the 8-month follow-up. The control group scored higher in eHealth literacy from baseline. The same pattern was identified regarding the parents' perceptions of internet usability and importance. Concerning satisfaction with CHC, accessibility, and support, the control group scored higher at baseline. Interestingly, the lines of the intervention and control groups crossed over at the 8-month follow-up.

**Conclusion:** Despite a limited outcome change, the results showed a tendency to benefit some parents. Our findings suggest that further evaluation, possibly with other more suitable measurements or questionnaires, an extended intervention period, and a larger sample, is necessary to understand the implications of these results fully.

## 1. Introduction

Internationally, parenting interventions have been shown to promote early child development effectively. A global systematic review and meta-analysis of parenting interventions delivered during the first 3 years of life found positive benefits for children's cognitive, language, motor, and socioemotional development, as well as attachment and reductions in behavioral problems [1]. Another scoping review mapped evidence on the implementation of parenting programs in real-world community settings, emphasizing the importance of fidelity, acceptability, and adaptations to improve program fit [2]. Additionally, parent-oriented mobile health interventions have significantly improved both parent and child health outcomes, showcasing the potential of digital tools to support parents outside of clinical settings [3].

In 2008, the Swedish government shifted its child health care strategy to emphasize support for the parental role, which led to the development of child health and guiding parental groups [4]. Recent studies have highlighted the effectiveness of these comprehensive programs in promoting child and family health [5, 6]. The Swedish child health care (CHC) program provides voluntary, no-cost services for children from birth to 5 years old, with 99% of Swedish parents participating in some form of CHC program. Sweden's extensive history of CHC has focused on prevention and parental support. Recent studies highlight the program's effectiveness and ongoing challenges in ensuring equitable access to health services [7]. Parent groups' content and structure have remained unchanged for years, raising questions about their relevance to parents' needs [8]. The experiences of the central child health services teams have highlighted the need for precise guidelines and stable financing to ensure the effective implementation of digital tools in child health services [7].

Child health nurses (CHNs) are expected to provide guidance, collaborate with other health care providers and social services, and tailor interventions to each family's situation. The national nurse-led CHC program includes home visits, growth and development assessments, and vaccination appointments. Medical check-ups are conducted by CHC nurses or in collaboration with general practitioners. The program offers parental groups and individualized parental support [4]. The program within CHC begins with a home visit within 1–3 weeks after the child's arrival following delivery, followed by regular meetings at CHC at specified intervals until the child reaches the age of 5. Of the approximately 12 visits, those at 1 month, 6 months, 12 months, and 2.5–3 years include medical check-ups with a general practitioner, while the others are nurse-led. Voluntary parental groups are offered for children aged 1–6 months, with about 40% participation [4, 8, 9]. Many regions in Sweden introduced digital parental groups during the pandemic. However, little is known about whether isolation and digital meetings, rather than face-to-face support, may have increased parental stress [10].

Stress is associated with various aspects of how individuals manage situations and relationships [11–13]. Parental stress can be defined as the stress that parents experience not only due to childrearing but also because of their social and environmental circumstances, responsibilities, and everyday life [14]. The stress a family experiences highly depends on the stressors, environment, and individuals involved. Therefore, supportive parental groups and contacts with parents who have similar experiences can reduce parental stress and increase their empowerment [15]. Modern parenting encompasses a diverse range of choices and approaches. Young parents are reported to experience heightened stress due to social media comparisons, which can exacerbate feelings of uncertainty and parental burnout [16, 17]. High parental stress does not significantly impact children's psychological well-being but does affect parental responsibilities [18].

In research, parental stress is gaining increased attention [19]. Hong et al. [20] and Wu and Xu [21] highlighted the need for comprehensive support to reduce parental stress, emphasizing the importance of professional guidance during the pandemic. Parental stress levels have remained high postpandemic, necessitating targeted public health interventions to address parent-specific stressors and provide long-term support [22]. Preventive support for stress can help parents handle future conflicts more effectively [23]. Despite the long-standing role of CHNs in parental education and support, the delivery methods and outcomes of parental groups have been inadequately evaluated [8, 24]. This study is aimed at evaluating the outcomes of a digital support intervention for parents, focusing on their stress, parental satisfaction, eHealth literacy, and satisfaction with CHC, as well as parents' perceptions of CHC accessibility and support.

## 2. Methods

### 2.1. Design

This study is a two-armed, cluster-randomized, prospective pilot intervention study with baseline, 4-month, and 8-month follow-up measurements.

### 2.2. Participants and Procedure

In two socioeconomically equal areas in northern Sweden, one CHC was randomly selected by lottery for intervention, offering digital support to an intervention group, and another was randomly selected as a control group. Parents from these CHCs were invited to participate in the study, which ran from autumn 2022 to late spring 2023. Informed consent letters were sent to the addresses of children registered at the respective CHCs. Parents of 528 children, aged 0–5 years, listed at the two CHCs, received a letter. In total, written informed consent was retrieved from 147 (28%) parents.

The intervention group (*n* = 75) received and returned a written informed consent letter. They also received written information about the study procedure, data collection, the available activities within the intervention, and instructions on digital registration. The control group (*n* = 72) also received and returned a written informed consent letter, and they were provided with information regarding the study and data collection procedures. Twenty-three participants did not respond to the baseline questionnaires, resulting in an 84.4% response rate. Of these, 65 participants were assigned to the intervention group and 59 to the control group. A description of the responders is given in Table 1.

### 2.3. Intervention

The digital intervention was implemented over an 8-month period, targeting parents of children aged 0–5 years. The intervention comprised several digital activities. Parental groups were offered, focusing on developmental stages at ages 1, 1.5, 2, 3, 4, and 5 years. During these digital 1-h sessions, the CHNs briefly presented general developmental milestones applicable to the child's age. Subsequently, parents engaged in discussions about their children's development and posed questions to one another, with the CHN facilitating as a moderator.

Furthermore, monthly 45-min digital groups were offered to learn about and discuss growth, breeding, and common child health issues, with topics such as breastfeeding, portion control, constipation, and eczema. The CHN briefly introduced the topics, for example, eczema, its causes, and factors that can aggravate or improve it. Following this, discussions continued regarding the parents' experiences with their child's eczema.

Parents could connect digitally with each other each week for open, hour-long meetings *without a set agenda*. During these sessions, the CHN acted as a moderator, offering information only when requested.

**Figure 1:**
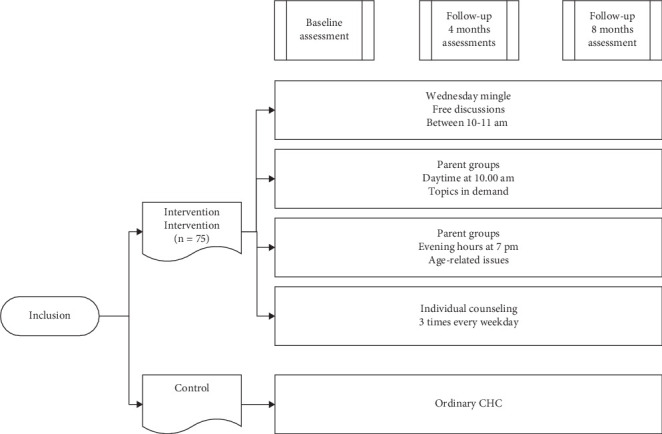
Description of the intervention and its components.

Additionally, three shorter, scheduled digital meetings were available from Monday to Friday, allowing parents to book *personal video meetings online* with the CHN. These meetings could concern a rash, wounds, or self-care advice regarding fever. The intervention is further described in Figure 1.

### 2.4. Measurements

The 18-item Parental Stress Scale (PSS), developed by Berry and Jones [25], assesses parental stress (10 items) and parental satisfaction (eight items). The scale has been psychometrically evaluated and found to be valid and reliable (Cronbach's alpha of 0.78 and 0.88, respectively) by Harding et al. [26]. They found that a two-factor solution best captured both stress and satisfaction related to the parenting role when two items (Items 8 and 14), one from each factor, were excluded, leaving 16 items. Parental stress consisted of nine items, and parental satisfaction comprised seven items. One of the original authors (Berry) permitted the translation of the instrument into Swedish. The PSS involves a 5-point Likert scale from 1 (*strongly disagree*) to 5 (*strongly agree*). A score is calculated by summing all items, resulting in a satisfaction subscale ranging from 7 to 35 and a stress subscale ranging from 9 to 45. Higher scores indicate greater parental satisfaction or stress [25, 26].

eHealth literacy refers to the ability to search, select, judge, and apply online health information to address or solve health problems and improve well-being. It encompasses a set of competencies and skills, including knowledge, comfort, and perceived ability to identify, evaluate, and apply electronic health information to health problems [27]. This assessment was conducted using the 10-item eHEALS scale developed by Norman and Skinner [28]. It has been validated in Swedish, demonstrating sufficient reliability and dependability, with a Cronbach's alpha of 0.94 [29]. The questions have five response options (1–5). The first two items, rated from *not at all useful* to *very useful*, assess participants' perceptions of the internet's usability and importance, analyzed separately. The remaining eight items (8–40), rated from *strongly disagree* to *strongly agree*, measure self-perceived knowledge of online searching and understanding health information, with higher scores indicating greater eHealth literacy. Parental satisfaction with CHC, accessibility in general, and if CHC was seen as supportive were answered by three items on a Visual Analogue Scale (VAS). An example of the question was, “To what extent do you think CHC is available to you as a parent?” The scale ranges from 0 to 100, with higher values indicating higher agreement.

### 2.5. Statistics

A power calculation was made based on the primary outcome measure of parental stress to demonstrate a statistically significant difference with a moderate effect between the intervention and control groups (cf. [30]). With a power of 0.8 and a significance level of 0.05, 77 participants were required in each group. However, we included 75 participants in the intervention group and 72 in the control group.

The means and standard deviations were reported for continuous variables, and continuous data were analyzed using the Mann–Whitney *U* test. The chi-squared (*χ*^2^) and Fisher's exact tests were used for dichotomous data, and normality was checked by the Shapiro–Wilk test. Repeated measures ANOVA was used to analyze changes over time. Level of education and distance to the CHC were included as covariates since the groups differed in these variables. Sphericity was checked utilizing Mauchly's test of sphericity. When appropriate, Huyn–Feldt's test was used. The effect size was calculated using a paired *t*-test with Cohen's *d* at baseline and 8 months as variables. According to Cohen [31], benchmarks for effect size are 0.2, 0.5, and 0.8, respectively, for small, medium, and large effect sizes. This study considered an effect size greater than 0.25 to be educationally significant [32]. The statistical program of JAMOVI v.2.3.24 was used for all analyses [33]. The Jamovi Project [33] (Jamovi Version 2.3) (computer software) was retrieved from https://www.jamovi.org.

### 2.6. Ethics

Participation was voluntary, and participants were informed by letter that they could withdraw their approval at any time. The data were stored in accordance with the data action plan and the guidelines of the Swedish Ethical Review Authority. All responses were labelled with a code, and the code list was securely locked in. The questionnaires were identifiable only through the code, and respondents were guaranteed confidentiality. The results are presented at the group level, meaning it is not possible to attribute data to a specific individual. The ethical application was approved by the Swedish Ethical Review Authority (No. 2021-00412).

## 3. Results

The parental satisfaction and stress between or within the intervention and control groups showed no significant changes from baseline to 8 months (Table 2). However, regarding eHealth literacy, significant differences were observed between groups, with both groups showing improvement from baseline to the 8-month follow-up (Figure 1). The control group scored higher in eHealth literacy at baseline and the 4- and 8-month follow-ups. The same pattern was identified regarding the parents' perceptions of internet usability and importance, which differed between groups in terms of the benefit of the control group, and a tendency for improvement over time was seen in both groups.

**Figure 2:**
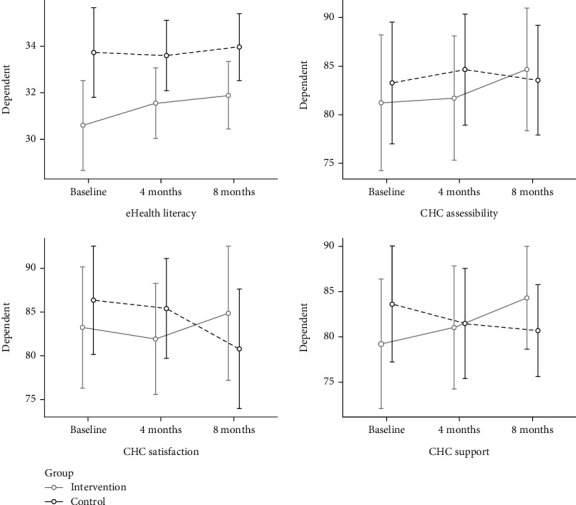
Estimates over time of eHealth literacy, as well as parental assessment of CHC accessibility, satisfaction with CHC, and CHS support.

Regarding satisfaction with CHC, accessibility of CHC, and views on CHC as supportive, the control group scored higher on all three measures at baseline. Furthermore, the lines between the intervention and control groups had crossed over at the 8-month follow-up (Figure 2).

## 4. Discussion

This study was aimed at evaluating the outcome of a digital support intervention for parents, focusing on its effects on their stress, parental satisfaction, eHealth literacy, and satisfaction with CHCs, as well as parents' perceptions of CHC accessibility and support. The result showed no significant differences in or between groups concerning parental stress during the intervention period. There was, however, an indication of improvements in eHealth literacy and satisfaction with CHC support and accessibility in the intervention group compared to the control group. On the contrary, the control group's satisfaction with CHC decreased during the 8-month intervention period. The results suggest the intervention did not have a noticeable, measurable direct effect, at least not on our chosen assessment variables or during the period we measured.

Although the present intervention was based on the parents' suggested topics, participation was still low. Lefèvre [9] argues that the low participation in parental groups is due to predetermined topics that the parents themselves do not determine. An optimistic interpretation of our study is that most Swedish parents may not require extensive support programs from CHC. In addition to CHC services, parents in Sweden have access to various other sources of support that can significantly aid in their parenting journey. One notable source is social services, particularly its preventive services, which offer educational programs to parents. These programs are designed to provide parents with the knowledge and skills needed to effectively manage the challenges of raising children [24]. An alternative interpretation is that the families are very occupied and suffer from a lack of time as both parents work full-time, often a year or two after the child is born. However, this does not necessarily mean they do not need support from the CHC. The factor of parental stress showed an effect size of 0.25 (cf. [32]) in the intervention group; this indicates that during the intervention (education), the parents began to gain insight into how to work towards decreasing their stress levels. However, the insights from the intervention have not yet fully clinically affected the parents, that is, they may not yet started to use the tools they received from the intervention in their daily lives. Regarding our measurements, parents rate themselves highly at baseline, so we cannot expect them to be any more satisfied than they already are. One possible explanation is that they are satisfied and grateful to be or become parents.

Only a few parents in the intervention group utilized digital support, and even fewer did so repeatedly. This is unfortunate, as parental groups can break isolation, foster extended friendships, and increase knowledge [8]. Lepistö et al. [34] argue that allowing parents to raise their concerns can prevent a lack of empowerment and self-efficacy and increase parental self-confidence. The intervention was offered to parents of children (0–5 years) who sometimes had several children at home. This aligns with Lagerberg and Magnusson's [35] results, which argue that CHN mainly focuses on first-time mothers, while mothers with more children are often left alone, resulting in stress. However, CHNs play a crucial role in supporting parents through reflection and self-awareness [17].

Armoiry et al. [36] compared the experiences of physical and digital encounters and concluded that they were equivalent. Both the face-to-face and digital encounters provided answers to the parental questions. If digital meetings are equivalent, as in Armoiry et al.'s review (2018), the economic aspect suggests a preference for digital meetings, which are more cost-effective in terms of travel and travel time. Additionally, supportive parental groups, comprising individuals with similar experiences, can help reduce parents' stress and increase their empowerment [15]. We argue that Sweden and other countries with large rural areas could benefit from digital interventions to create more equal conditions based on distance and other complicating factors, such as various family constellations, economic opportunities, and transportation costs.

Furthermore, one study highlighted the need for more rigorous research on cultural adaptation and implementation practices [37]. Lefèvre et al. [9] suggested that parental groups would achieve higher participation rates if they were more parent-driven and if CHNs were more confident in facilitating them. Similarly, Gonzalez et al. [38] argued that individual engagement in network groups is stronger when the network is tailored to users' needs. Engagement is enhanced when information is conveyed at the user's level and learning opportunities are provided. Hussain and Tait [39] found that parents, especially those with children with disabilities, benefit from digital support to reduce their stress since they often lack targeted support in traditional settings.

Our goal was to tailor the intervention to meet the needs of parents. A study by Valan et al. [40] emphasizes the importance of ensuring good accessibility, allowing for the quick booking of appointments, and the prompt provision of responses. In contrast to these results, the participation rate was low in the present intervention. However, another study shows that digital consultation opportunities simplify parenting and increase security, as parents can stay home with their children while still accessing care [41]. A review mapping the evidence on evidence-based parenting programs emphasizes the need for high-quality implementation and calls for more consistent reporting to maximize public health impact [2]. Recent research suggests that understanding the conditions under which parenting programs succeed or fail can enhance effective implementation and benefit children and families [2, 42].

### 4.1. Methodological Considerations

One strength of this study was that the use of repeated-measures ANOVA demonstrated the effect of the intervention over time and revealed the differences between the two groups, intervention and control. However, we found that the intervention had limited effects for several reasons. Firstly, the control group scored higher in eHealth literacy, internet use, and satisfaction with CHC support and accessibility at baseline. They also lived closer to the CHC than the intervention group and had higher education levels and more extensive parenting experience, as indicated by having more children. Secondly, there was a relatively high dropout rate, potentially due to participants feeling that they lacked sufficient time to engage during the preschool years. Thirdly, only a small number of parents utilized the intervention's content. Of the 75 participants, only 22 engaged in one or more activities, while 53 chose not to participate, and just five parents attended multiple activities. This means that many questionnaires were likely completed without the parents engaging in the activities, leading to a careful interpretation of the findings. The intervention's effect on parental stress and eHealth literacy was not statistically significant between groups, possibly due to the short intervention period and low engagement. Another weakness was the choice of outcome variables; measures like empowerment or self-efficacy might have yielded different insights. Despite the nonsignificant results, this pilot study provides valuable information about the digital support intervention.

## 5. Conclusion

The intervention needs further development, testing in larger contexts, and qualitative evaluation through interviews with parents and CHC nurses. Although the outcome change was limited, results indicated some potential benefits for the parents. These findings suggest the need for further evaluation using different outcome measures or questionnaires, an extended intervention period, and a larger sample size. Additional studies should examine how CHC nurses perceive the extra workload from digital support and assess its health-economic value. To enhance understanding of parental perspectives, their inclusion in future studies is vital. Evaluating a similar intervention may benefit from qualitative methods, as quantitative questionnaires often overlook crucial experiences and perspectives.

## Figures and Tables

**Table 1:** Description of the respondents.

	**Total (** **n** = 124^**a**^**)**	**Intervention group (** **n** = 65**)**	**Control group (** **n** = 59**)**	**p** ** value**
Age (mean, SD)	36.0 (5.53)	35.2 (6.25)	36.8 (4.53)	0.072
Female (*n*, %)	90 (72.6)	47 (72.3)	43 (72.9)	0.943
Education (*n*, %)				0.006
High school	47 (37.9)	32 (49.2)	15 (25.4)	
University	77 (62.1)	33 (50.8)	44 (74.6)	
Marital status (*n*, %)				0.347
Cohabiting/married	117 (95.1)	62 (96.9)	55 (93.2)	
Single	6 (4.9)	2 (3.1)	4 (6.8)	
Number of children (*n*, %)				0.085
1 child	36 (29.0)	24 (36.9)	12 (20.3)	
2 children	54 (43.5)	25 (38.5)	29 (49.2)	
3 children	29 (23.4)	12 (18.5)	17 (28.8)	
More than 3 children	5 (4.0)	4 (6.2)	1 (1.7)	
Distance to CHC in kilometers (mean, SD)	6.42 (7.71)	8.82 (9.23)	3.36 (4.46)	< 0.001

^a^Missing (*n* = 23).

**Table 2:** Changes within and between groups, intervention versus control, at baseline, and 4- and 8-month follow-up.

**Factors (range)**	**Baseline** **Mean (SD)** **(** **n** = 65** vs. 59)**	**4 months** **Mean (SD)** **(** **n** = 44** vs. 46)**	**8 months** **Mean (SD)** **(** **n** = 41** vs. 47)**	**p** ** value** **Within groups** **(** **n** = 32/40**)**	**p** ** value** **Between groups** **(** **n** = 32/40**)**	**Effect-size Cohen's ** **d**
Parental satisfaction (7–35)				0.882	0.076	
Intervention	32.88 (2.63)	32.55 (2.54)	32.51 (2.78)			0.02
Control	33.41 (1.55)	33.33 (1.94)	33.34 (1.81)			0.02
Parental stress (9–45)				0.452	0.351	
Intervention	21.83 (4.83)	22.20 (4.54)	22.56 (4.83)			0.25
Control	21.00 (5.28)	21.11 (5.24)	21.51 (5.20)			0.09
eHEALS (8–40)				0.492	0.019	
Intervention	30.69 (6.33)	32.02 (5.66)	31.83 (4.88)			0.29
Control	33.02 (4.22)	34.26 (4.06)	34.36 (3.90)			0.20
Internet usability (1–5)				0.727	0.002	
Intervention	3.72 (0.93)	3.91 (0.77)	3.88 (0.81)			0.11
Control	4.10 (0.66)	4.30 (0.66)	4.36 (0.57)			0.29
Internet importance (1–5)				0.899	0.004	
Intervention	3.95 (0.91)	4.11 (0.89)	3.98 (1.01)			0.04
Control	4.36 (0.64)	4.59 (0.58)	4.51 (0.51)			0.14
CHC accessibility (0–100)				0.480	0.742	
Intervention	83.22 (21.54)	82.00 (19.84)	82.39 (21.61)			0.15
Control	84.12 (16.70)	85.09 (14.68)	83.39 (16.01)			0.00
Satisfaction with CHC (0–100)				0.101	0.838	
Intervention	85.02 (19.03)	82.32 (18.73)	83.44 (21.00)			0.11
Control	88.03 (14.60)	86.46 (15.91)	81.09 (22.07)			0.31
CHC as support (0–100)				0.221	0.910	
Intervention	81.19 (22.73)	81.45 (21.25)	83.59 (15.61)			0.25
Control	82.71 (16.89)	82.02 (16.00)	80.45 (16.64)			0.09

## Data Availability

Due to ethical and privacy reasons, data are not publicly available. Data will be accessible from the corresponding author upon reasonable request.
